# A chromosome level genome assembly of *Pseudoroegneria Libanotica* reveals a key *Kcs* gene involves in the cuticular wax elongation for drought resistance

**DOI:** 10.1186/s12864-024-10140-5

**Published:** 2024-03-06

**Authors:** Xingguang Zhai, Dandan Wu, Chen Chen, Xunzhe Yang, Shaobo Cheng, Lina Sha, Shuhan Deng, Yiran Cheng, Xing Fan, Houyang Kang, Yi Wang, Dengcai Liu, Yonghong Zhou, Haiqin Zhang

**Affiliations:** 1 State Key Laboratory of Crop Gene Exploration and Utilization in Southwest China, Sichuan Agricultural University, Chengdu, Sichuan 611130 China; 2 Triticeae Research Institute, Sichuan Agricultural University, Chengdu, Sichuan 611130 China; 3 College of Grassland Science and Technology, Sichuan Agricultural University, Chengdu, Sichuan 611130 China; 4 Glbizzia Biosciences Co., Ltd, Liandong U Valley, Huatuo Road 50, Daxing, Beijing, 102600 China

**Keywords:** Genome assemble, St genome, Triticeae, Comparative analyses, Cuticular wax, Drought resistance

## Abstract

**Background:**

The genus Pseudoroegneria (Nevski) Löve (Triticeae, Poaceae), whose genome symbol was designed as “St”, accounts for more than 60% of perennial Triticeae species. The diploid species Psudoroegneria libanotica (2n = 14) contains the most ancient St genome, exhibited strong drought resistance, and was morphologically covered by cuticular wax on the aerial part. Therefore, the St-genome sequencing data could provide fundamental information for studies of genome evolution and reveal its mechanisms of cuticular wax and drought resistance.

**Results:**

In this study, we reported the chromosome-level genome assembly for the St genome of Pse. libanotica, with a total size of 2.99 Gb. 46,369 protein-coding genes annotated and 71.62% was repeat sequences. Comparative analyses revealed that the genus Pseudoroegneria diverged during the middle and late Miocene. During this period, unique genes, gene family expansion, and contraction in Pse. libanotica were enriched in biotic and abiotic stresses, such as fatty acid biosynthesis which may greatly contribute to its drought adaption. Furthermore, we investigated genes associated with the cuticular wax formation and water deficit and found a new Kcs gene evm.TU.CTG175.54. It plays a critical role in the very long chain fatty acid (VLCFA) elongation from C18 to C26 in Pse. libanotica. The function needs more evidence to be verified.

**Conclusions:**

We sequenced and assembled the St genome in Triticeae and discovered a new KCS gene that plays a role in wax extension to cope with drought. Our study lays a foundation for the genome diversification of Triticeae species and deciphers cuticular wax formation genes involved in drought resistance.

**Supplementary Information:**

The online version contains supplementary material available at 10.1186/s12864-024-10140-5.

## Background

The Triticeae tribe (Poaceae), includes economically important annual crops (e.g., wheat, rye, and barley) and crucial perennial forage grasses (e.g., *Roegneria*, *Agropyron*, *Leymus*, and *Pseudoroegneria*) [1]. The genus *Pseudoroegneria* consists of six diploids (2n = 2x = 14, StSt) and nine autotetraploid species (2n = 4x = 28, StStStSt) [2]. *Pseudoroegneria* was built around one genome designated as St, which is one of the most important basic genomes (St, E, H, P, W, and Ns) in perennial Triticeae. The St genome is the donor genome of species in eight polyploid genera, including *Roegneria* (StY), *Douglasdeweya* (StP), *Elymus* (StH), *Thinopyrum* (StE), *Campeiostachys* (StYH), *Anthosachne* (StYW), *Kengyilia* (StYP), and *Pascopyrum* (StHNsXm) [2, 3]. At present, the St-containing species were utilized for wheat breeding, such as wheat-*Thinopyrum poticum* (StStEEE) translocation line with stripe rust resistance [4], Wheat-*Elymus repens* (StStH) 3D/3St translocation line with highly resistance to Fusarium head blight (FHB) [5]. Recently, a new resistance locus *FhbRc1* was mapped to the distal region of chromosome 7S^c^L of *Roegneria ciliaris* [6, 7]. In addition, high grass production, excellent disease, and saline-alkali resistance characteristics make the St-containing species to be the ideal germplasm for selecting forage varieties and ecological restoration [8, 9]. Thus, the St genome not only accounts for extensive perennial speciation in Triticeae, but also is prominent for forage and crop breeding.

More and more efforts involved the origin of the St genome and genetic relationships among the Triticeae species. Molecular data based on several single-copy genes or transcriptome analysis estimated that the genus *Pseudoroegneria* is more ancient than the *Triticum*/*Aegilops* group but younger than the genus *Psathyrostachys* (genome symbol was designed as Ns) [10, 11]. Genomic affinity suggested that the St genome was closely related to the J (= E) genome of *Thinopyrum elongatum*, and diverged from H (*Hordeum bogdanii*), D sub-genome, then followed by B and A sub-genome in common wheat [12, 13]. Recently, Wang et al. (2020) mapped 14 linkage groups (LGs) and identified seven homologous groups of the St genome, and revealed the genome shared homology between the St and ABD, and H genome (35% and 24%, respectively) [14]. Karyotype comparative analyses revealed a highly conserved cytogenetic collinearity between the St genome and the common wheat genome, except for a well-known 4 A chromosome translocation [15]. These studies provide partly St genome information, nevertheless, the whole genome homoeology and genetic relationship between the St genome and the published Triticeae species from whole genome level have not been studied.

*Pseudoroegneria* plants are predominately cool-season and drought-tolerant grasses, which are distributed in arid or semi-arid areas. The diploid *Pse. libanotica* grows in the lithoid slopes of Lebanon, Iraq, and the north of Iran [2]. Molecular and phylogeny studies of Triticeae species showed that *Pse. libanotica* has earlier speciation at phylogenetic tree, implying probably a more ancient St genome than diploid *Pseudoroegneria* species from Eastern Europe (*Pse. strigosa*), East Asia (*Pse. stipifolia*), and North America (*Pse. spicata*) [16, 17]. Morphologically, *Pse. libanotica* is covered by thickened cuticular wax on the aerial part and it has been reported that the cuticular waxes is essential to prevent non-stomata water loss in *Pseudoroegneria* [8, 18]. Thus, characterizing the St genome of *Pse. libanotica* might reveal the regulation of cuticular wax biosynthesis in response to water deficit.

The regulation of cuticle deposition in response to drought stress was found in model species, crops, and important economic species such as *Arabidopsis thaliana*, *Oryza sativa*, wheat (*Triticum aestivum*), maize, soybean (*Glycine max*), sesame (*Sesamum indicum*), sorghum, oats (*Avena sativa*), *Medicago sativa*, cotton (*Gossypium hirsutum*), tree tobacco (*Nicotiana glauca*), and pine (*Pinus palustris*) [19–21]. Cuticular waxes mostly comprise very long chain fatty acids (VLCFAs) and their derivatives, including alkanes, wax esters, branched alkanes, primary alcohols, alkenes, secondary alcohols, aldehydes ketones, and unsaturated fatty alcohols, as well as cyclic compounds including terpenoids and metabolites, such as sterols and flavonoids. Multiple genes involved the cuticular wax biosynthesis have been investigated. The plastid Acetyl-CoA carboxylases are responsible for *de novo* fatty acid biosynthesis (up to C16:0, C16:1, and C18:1) [22, 23]. Further, the long-chain fatty acids (C16–C18) are exported to the cytosol after the hydrolysis of ACPs by acyl-ACP thioesterases [24]. These 3-ketoacyl-CoA synthases (KCS) isoforms are involved in fatty acid elongation [25]. Some cytochrome P450s have been reported to be involved in plant cuticle wax synthesis as well [26, 27]. Eceriferum (CER) genes affect different steps of the wax biosynthesis pathway including primary alcohol forming, aldehydes, and alkanes synthesis [28]. Researchers investigated distinctive cuticular waxes among different plant species, tissues, and organs, even in different growth and developmental stages [29]. Novel genes involved in cuticular wax biosynthesis may be practically used as valuable genetic resources to improve drought tolerance in crop breeding [30]. However, drought-induced wax biosynthesis has been seldomly investigated in wild germplasms in Triticeae.

Given the importance of the St genome in Triticeae and the excellent drought resistance traits of the *Pseudoroegneria* species, the diploid wild species *Pse. libanotica* was performed whole-genome sequencing in this study. We sequence and assemble the chromosome-scale reference St genome to (1) identify the genetic relationship, especially among Triticeae species using whole genome comparative analysis; (2) elucidate the mechanism of fatty acid biosynthesis in *Pse. libanotica* under drought stress. Those results will provide solid information for the evolution of Triticeae species, and facility better germplasm utilization.

## Results

### Genome assembly, quality evaluation, and annotation

Before the genome *de novo* assembly, a genome size survey of *Pse. libanotica* (PI 228,392) based on flow cytometry and k-mer statistics is about 3,273.28 Mb (1 C) and 3,048.18 Mb, respectively (Fig. S1). The genome of *Pse. libanotica* was *de novo* assembled by integrating ~ 191 Gb (64×) Illumina short paired-end reads, ~ 440 Gb (sequencing depth 147×) Nanopore sequencing data, and ~ 330 Gb (110×) high-throughput chromosome conformation capture (Hi-C) data to generate the V1.0 assembly. For draft assembly improvement, we conducted three rounds of self-correction for Nanopore data and Illumina correction and generated a chromosome-level assembly of *Pse. libanotica*. The assembly sequence comprised 2.99 Gb of genome data, with a contig N50 of 0.96 Mb and a super-scaffold N50 of 398.55 Mb, accounting for 96.45% of the estimated genome size with 45.07% GC content and 1.34% heterozygous (Table 1; Table S1 and S2; Fig. S2 and S3). Of the 2.99 Gb scaffold sequences, there are 2.75 Gb (92.09%) totaling 4060 scaffolds were anchored to seven super-scaffolds (chromosomes) using the Hi-C platform. The number of chromosome-scale super scaffolds is consistent with the species’ determined chromosome number of 7. The shortest chromosome is Chr4 (327.30 Mb), which contains 462 scaffolds, and the longest chromosome is Chr2 (464.04 Mb), which contains 660 scaffolds (Fig. S4; Table S3). The assembly accuracy and the qv were 23.23, which indicated the reliability of the assembly genome.

**Table 1:** Overview of genome assembly and gene annotation for *Pse. libanotica*

Assembly characteristics	Values
Estimated genome size	3.10 Gb
Assembled genome size	2.99 Gb
Total length of contigs	2.99 Gb
N50 length of contigs	0.96 Mb
Total number of contigs	7,391
N50 length of super scaffolds	398.55 Mb
Number of annotated high-confidence genes	46,369
Percentage of repeat sequences	71.62%
Complete BUSCOs	95.20%
Fragmented BUSCOs	1.20%
Missed BUSCOs	3.60%

The long terminal repeat (LTR) Assembly Index (LAI), which evaluates the contiguity of intergenic and repetitive regions of genome assemblies based on the intactness of LTR retrotransposons (LTR-RTs) [31], of the *Pse. libanotica* genome assembly was 13.20 (Table S4), reaching to the criterion of reference quality. Further, we conducted CEGMA [32] and BUSCO [33] to evaluate the integrity and base accuracy of the assembled *Pse. libanotica* genome. CEGMA showed that the assembled genome completely covered 228 (91.94%) of the 248 core genes, and partially covered 11 core genes. Less than 4% of the core genes were not detected. BUSCO displayed that 95.2% of the 1440 single-copy genes were homologous sequences in Triticeae species (Table S5). The draft assembly was further evaluated by mapping short high-quality reads into the assembled genome. The mapping rate was 98.95%, with 58.95% of the average sequencing depth (Table S6). In *Pse. libanotica*, 151,872 expressed sequence tag (EST) sequences were mapped to the genome with > 95% identity, in which 132,240 (87.10%) were aligned to the reference genome with > 87% coverage (Table S7). Collectively, these data showed the high coverage of the assembled St genome.

**Fig. 1:**
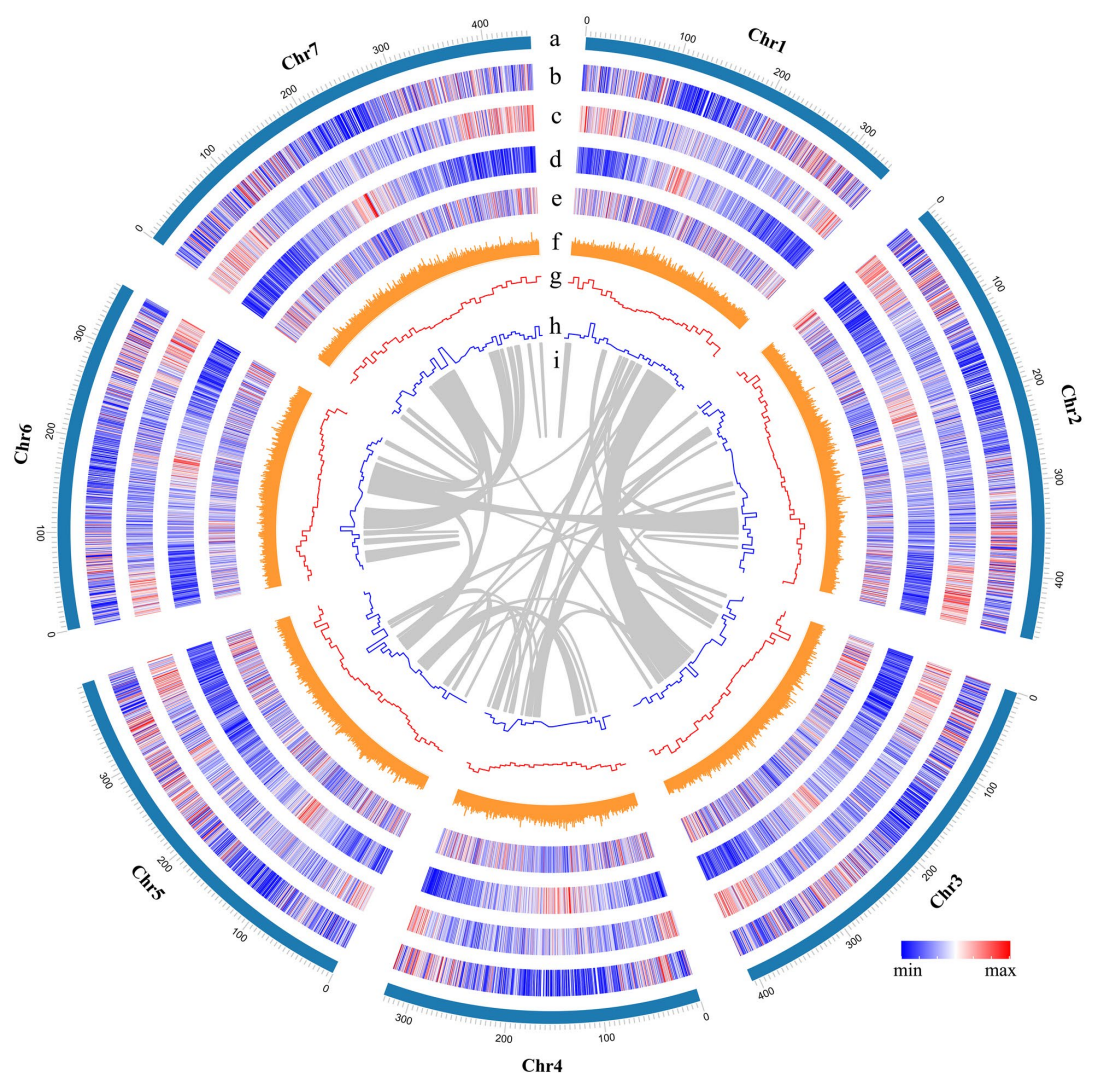
Overview of the *Pse. libanotica* PI 228,392 genome. The tracks indicate (moving inwards): (a) Chromosomes (Chr1 ~ 7) name and size, (b) Gene density, (c) repeat density (window size of 500 kb), (d) LTR-Gypsy (window size of 500 kb), (e) LTR-Copia (window size of 500 kb), (f) GC content (%), (g) Expansion gene (window size of 10 Mb), (h) Contraction gene (window size of 10 Mb), (i) Chromosome homologous relationship

A total of 46,369 protein-coding genes were identified, 82.89% of which had functional annotations, and 67.16% were annotated with functional domain using eggNOG database (v5.0) (Fig. 1; Tables S8, S9). Moreover, we identified 1,483 transfer RNAs, 18,438 miRNAs, 1,427 small nuclear RNAs, and 473 ribosomal RNAs (Table S10). Repeat sequences comprised 71.62% of the assembled genome, in which the long terminal repeat-retrotransposon (LTRs) were the most abundant repeat type, including two ubiquitous classes Gypsy and Copia. Meanwhile, the DNA retrotransposon, short interspersed nuclear elements (SINEs), and long interspersed nuclear elements (LINEs) had the lowest proportion in the final assembly (Table 2, S11). The Gypsy family density was increased from the telomere to the centromere, while the Copia family was uniformly distributed along the seven chromosomes (Fig. 1).

**Table 2:** Statistics of transposable elements in *Pse. libanotica* genome sequences

	Denovo+Repbase Length(bp)	% in Genome	TE proteins Length(bp)	% in Genome	Combined Tes Length(bp)	% in Genome
DNA	80,757,639	2.70	49,637,759	1.66	128,642,083	4.30
LINE	2,196,166	0.07	33,738,564	1.13	35,319,985	1.18
SINE	1,570,054	0.05	0	0	1,570,054	0.05
LTR	1,952,684,497	65.32	391,751,832	13.1	1,977,516,340	66.15
Unknown	9,318,886	0.31	120	0.000004	9,319,006	0.31
Total	2,045,135,021	68.41	475,109,220	15.89	2,105,455,067	70.43

### Phylogenetic evolution, whole-genome duplication, and genome synteny

**Fig. 2:**
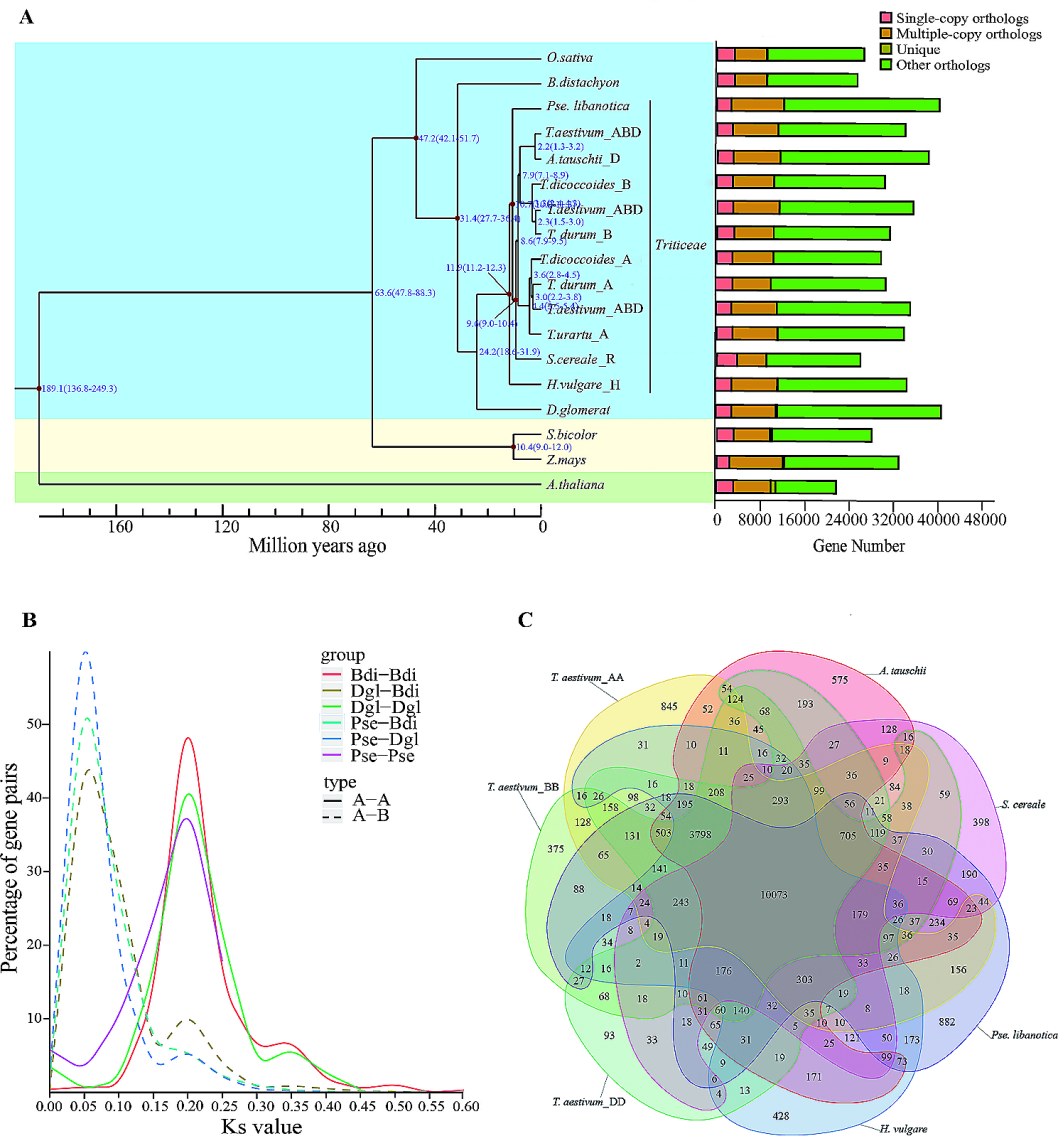
Gene family and genome evolution of *Pse. libanotica*. **A**: The left panel includes the estimation of divergence time of *Pse. libanotica* and *O. sativa*, *B. distachyon*, *T. aestivum*, *A. tauschii*, *T. durum*, *T. dicoccoides*, *T. urartu*, *S. cereale*, *H. vulgare*, *Z. mays* and *(A) thaliana*. The right panel displays the distribution of single-copy, multiple-copy, unique and other orthologues. **B**: Distribution of the ks values of the best reciprocal BLASTP hits in the genomes of *Pse. libanotica* (Pse), *D. glomerata* (Dgl) and *(B) distachyon* (Bdi). **C**: The number of gene families shared among five Triticeae species genomes shown in Venn diagrams. The overlap between the circles indicates gene families shared between species, and the numbers refer to the number of gene families

To explore the phylogeny and divergence of the St genome, we reconstructed the phylogenetic tree using single-copy ortholog genes shared by 14 Poaceae plants (Fig. 2A). *Sorghum bicolor* and *Zea mays* were C_4_ plants and diverged firstly in the phylogenetic tree, followed by *O. sativa*, *Brachypodium distachyon*, and *Dactylis glomerat*. Triticeae species were clustered into one monophyletic group (*Aegilops tauschii*, *Hordeum vulgare*, *Secale cereale*, *T. aestivum*, *T. durum*, *T. dicoccoides*, *T. urartu*). The *Pse. libanotica* diverged ~ 10.7 million years ago (Mya) after *H. vulgare* (~ 11.9 Mya), while the *S. cereale* and the genus *Triticum* separated ~ 9.6 Mya. To clarify whole-genome duplication in *Pse. libanotica*, synonymous substitutions (Ks) were calculated in *Pse. libanotica*, *D. glomerat*, and *B. distachyon*. The peak Ks at 0.20 which occurred after the divergence peak at 0.05 between *Pse. libanotica* and *D. glomerat* and 0.05 between *Pse. libanotica* and *B. distachyon*, indicating that a whole-genome duplication event occurred in the common ancestor of Poaceae (Fig. 2B).

Orthologous genes in *Pse. libanotica*, *T. aestivum*, *T. urartu*, *H. vulgare*, and *D. glomerat* were analyzed to illustrate chromosome collinearity and derivation (Fig. S5). *Pse. libanotica* exhibited conserved chromosome homoeologous collinearity with A genome, B-subgenome, D-subgenome, and H genome, and varied with *D. glomerat.* Interestingly, the 4St in *Pse. libanotica* and the 4 A chromosome of *T. urartu* displayed high consistency. Nevertheless, a pericentric inversion was observed in the 4 A chromosome, and a 4AL (long arm)-5AL-7BS (short arm) translocation in the common wheat. The St genome and the *D. glomerat* displayed chaotic chromosome collinearity. The 2St and 3St chromosomes mainly matched to the 1 and 5 chromosomes of *D. glomerat*, the 5St had syntenic to the 3 and 4 chromosomes of *D. glomerat*, and the 7 St was syntenic to the 3 and 7 chromosomes of *D. glomerat* (Fig. S5). Thus, gene order and location varied among Poaceae species after divergence, conserved among diploid Triticeae species, and partly rearranged during the polyploidization of Triticeae.

### Unique gene families, contract and expanded gene families involved in cuticular wax biosynthesis in *pse. Libanotica*

To characterize the St genome, unique gene families, contract and expanded gene families were analyzed in the St genome. In total, 10,073 shared gene families were identified in published Triticeae species and *B. distachyon*, whereas 882 gene families were specific to *Pse. libanotica* (Fig. 2C; Table S1). Unique gene families in *Pse. libanotica* were mainly involved in MAPK signaling pathway, ABC transport, and cuticular wax biosynthetic pathways (Fig. S6; Table S12, S13). Compared with the most recent common ancestor (MRCA), *Pse. libanotica* contained 3,558 expanded and 7,007 contracted genes (Table S14). There showed no distribution preference bias of expanded or contracted gene location among seven chromosomes, whereas GO term analysis revealed that most expanded or contracted genes were enriched in protein binding molecular functions including fatty acid binding. KEGG pathway enrichment analysis showed that the expanded gens were in five pathways: cutin, suberine, and wax biosynthesis, sesquiterpenoid and triterpenoid biosynthesis, flavone and flavonol biosynthesis, and benzoxazinoid biosynthesis (Table S15, S16). In summary, unique gene families, expanded and contracted gene families in the St genome are clustered in the cutin, suberine, and wax biosynthesis pathways.

### Fatty acid-related genes under drought stress in *pse. Libanotica*

To identify candidate genetic regions associated with fatty acids and their derivatives metabolism pathway, we performed 28 d drought stress treatment without irrigation and conducted transcriptome sequencing (6 Gb/sample) on each treatment. Every seven days was taken as a treatment 7 d, 14 d, 21 d, 28 d, and each treatment has three biology repeats. Total reads and mapping ratio were listed in Table S17. The number of differentially expressed genes (DEGs) under drought conditions at 7d, 14d, 21d, and 28d were selected using criteria| log2FC| ≥ 1 and adjusted q-value < 0.05 (Table S18). We found that DGEs were largely expressed at 14 d drought treatment. In total, 1,010 co-expressed DEGs were shared at four treatments (Fig. S7A, S7B). GO enrichment analysis showed that 1,190 GO terms were assigned to the 2,300 DEGs that responded to drought treatment (Fig. S8). KEGG enrichment analysis showed that DEGs were mainly involved in the biosynthesis of unsaturated fatty acids, fatty acid metabolism and the elongation process (Fig. S9). Comparing the DEGs with expansion and contraction genes, 1,576 DEGs (9.25%) were found in the expansion genes, whereas 61 DEGs (29.35%) were found in the contraction genes.

**Fig. 3:**
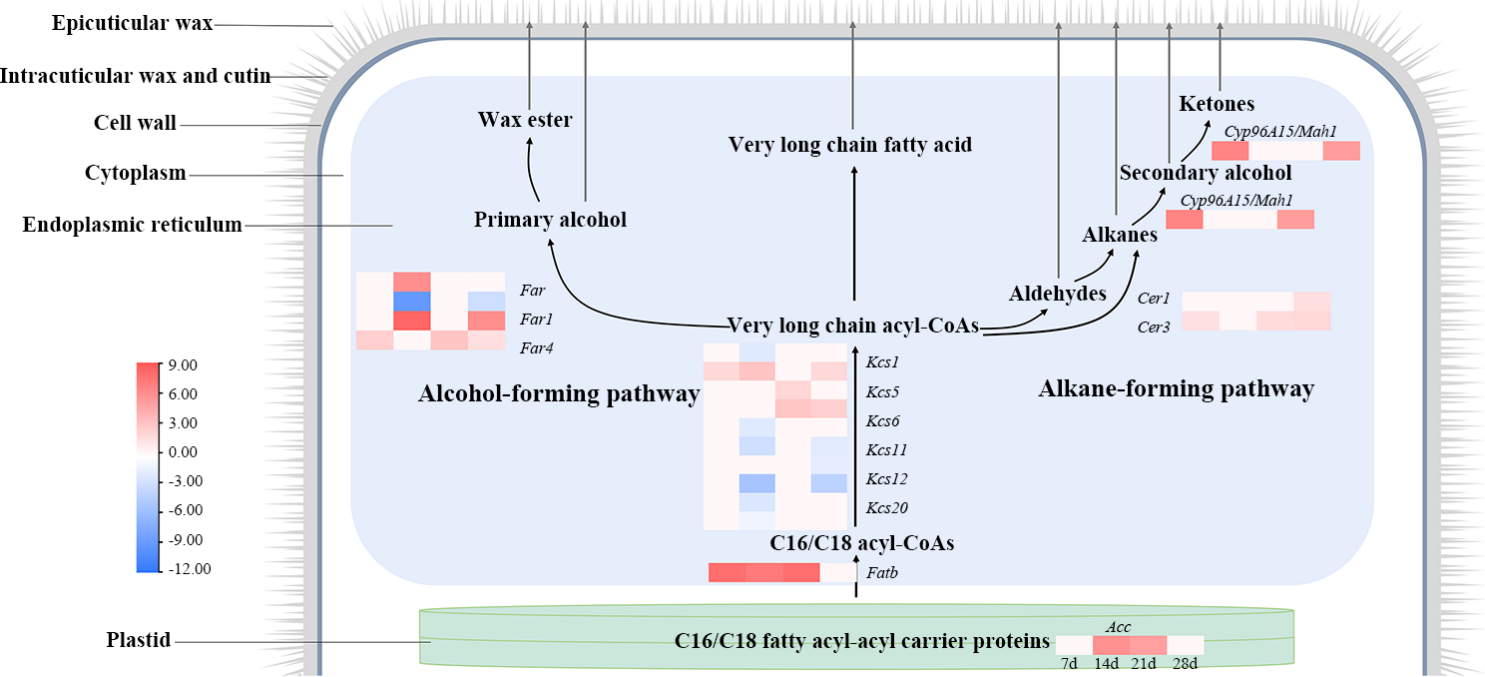
Cuticular wax biosynthetic pathway (www.kegg.jp/kegg/kegg1.html) related genes in *Pse. libanotica* under drought stress. Cuticular wax are synthesized and secreted in the endoplasmic reticulum (ER). First, long-chain fatty acids (C16–C18) are synthesized in the plastids. Second, the saturated C16 and C18 fatty acyl-CoA precursors are elongated to very long chain fatty acyl-coenzyme As (VLCFA-CoAs) in ER. Once the VLC-acyl-CoAs are synthesized, they can be further released as free VLCFAs that can either be directly exported as cuticular waxes, or undergo further modifications in the via either the alkane-forming pathway or the alcohol-forming pathway

In total, we identified 14 significantly different expression genes (*ACC*, *FATB*, *FACR*, *FACR1*, *FACR4*, *KCS1*, *KCS5*, *KCS6*, *KCS11*, *KCS12*, *KCS20*, *CER1*, *CER3* and *CYP96A15*) including 19 transcripts that directedly participated in the fatty acid biosynthesis pathway under 28 days of drought stress (Table S19). Phylogeny tree of the transcripts corresponding genes, involving in wax biosynthesis, displayed that *Kcs* gene family, *Facr* gene family and *Cer* gene family were formed subclade respectively (Fig. S10). *Fatb* was annotated as a novel gene (novel.7326), thus, it was absent in the phylogenetic tree. During the *de novo* fatty acid biosynthesis, the *Acc* and *Fatb* were upregulated during 21-day water deficit. During fatty acid elongation, except for two transcripts of *Kcs5* and *evm.TU.CTG175.54*, other *Kcs* genes (*Kcs1*, *Kcs6*, *Kcs11*, *Kcs12*, and *Kcs20*) were significantly downregulated. Genes catalyzed VCLFAs derivatives in alcohol-forming pathway (*Far*, *Far1*, and *Far4*) and alkane-forming pathway (*Cer1*, *Cer3*/*Cytb5*, *Cyp96a15*/*Mah1*) were upregulated apart from one of the *Far1* transcript *evm.TU.CTG5611.2* (Fig. 3). In summary, we speculated that only *Kcs5* and *evm.TU.CTG175.54* were responsible for VCLFA elongation in *Pse. libanotica*.

### Characterization of the *evm.TU.CTG175.54* gene related to VCLFA elongation

**Fig. 4:**
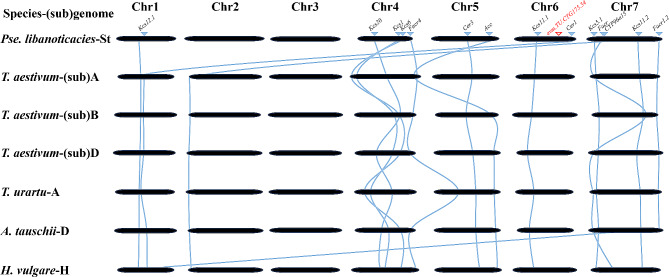
Microsynteny analysis between fatty acid biosynthesis candidate genes loci and their respective synteny counterparts in common *T. aestivum* (ABD), *T. urartu* (A), *A. tauschii* (D) and *H. vulgare* (H). The syntenic genes are connected by colored lines. The *evm.TU.CTG175.54* were displayed in red line. The arrowhead indicates genes resided in the *Pse. libanotica* locus, and gene names are above the arrowhead. Corresponding syntenic gene names are displayed below

To characterize the *evm.TU.CTG175.54*, synteny analysis among major Triticeae species (*T. aestivum*, *T. urartu*, *A. tauschii*, and *H. vulgare*) were conducted. Candidate genes presented ortholog among major Triticeae genomes, however, no orthologs of the *evm.TU.CTG175.54* were found (Fig. 4). We aligned the cDNA in the National Center for Biotechnology Information (https://blast.ncbi.nlm.nih.gov/Blast.cgi, NCBI database), and the result showed that it was *Kcs5* like and/or *Kcs6* like in Poaceae species, such as *T. aestivum*, *A*. *tauschii*, *H. vulgare*, *Lolium rigidum*, *Z. mays*, *O. sativa*, and *Panicum virgatum*, etc. Comparative analysis of cDNA sequence showed that 86.92% and 77.23% similarity shared between *evm.TU.CTG175.54* and *Kcs5* and *Kcs6*, respectively.

**Fig. 5:**
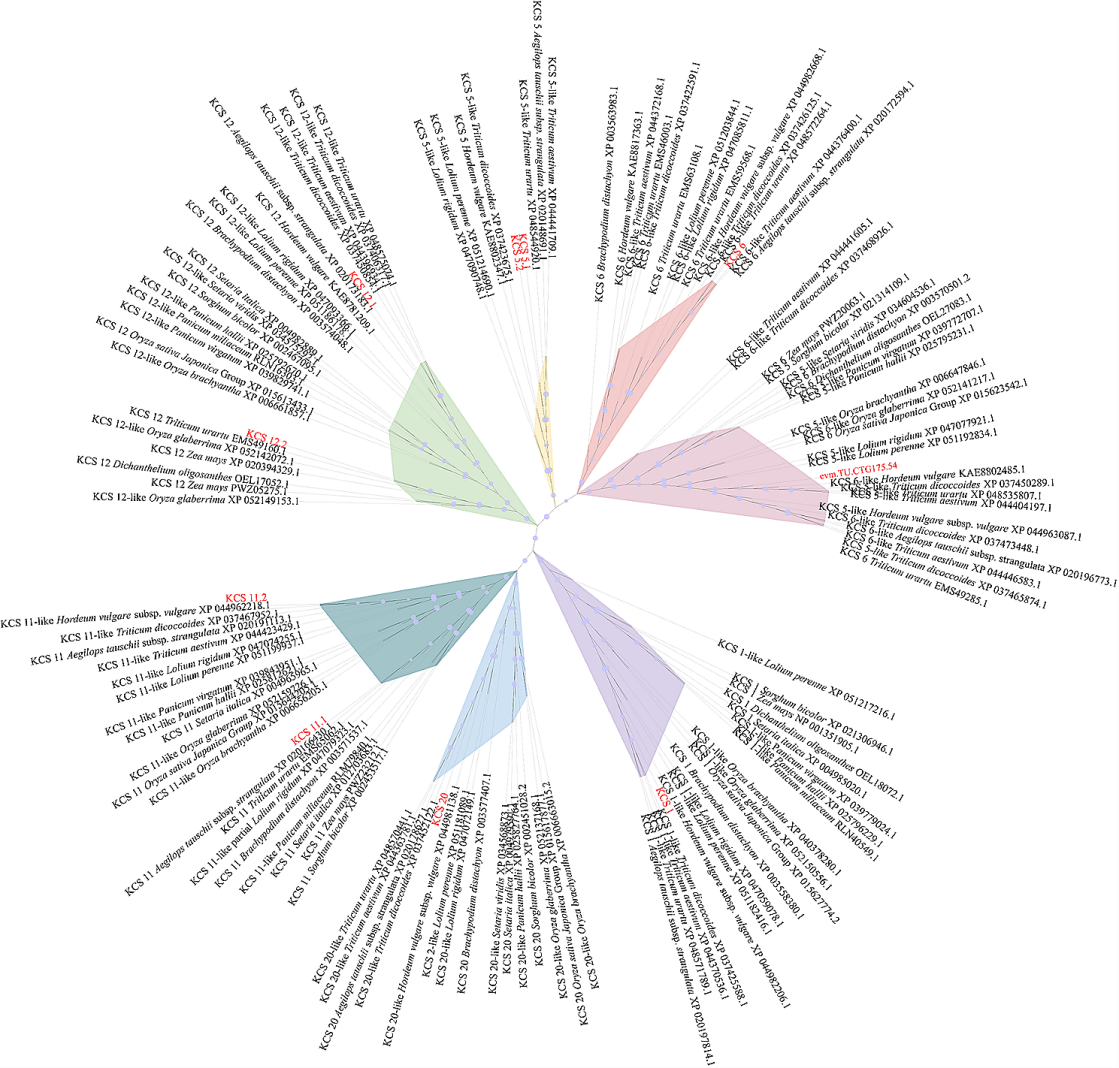
Maximum likelihood tree derived from 132 KCS protein sequences among Poaceae. KCS proteins in this research were presented in red, while KCS proteins downloaded from NCBI were nominated as ‘KCS protein + species + protein entry’

The phylogenetic tree based on 132 KCS proteins in Poaceae displayed that the *evm.TU.CTG175.54* distributed in a subclade including KCS5 or KCS6 similar proteins, while other KCS proteins (KCS1, KCS5, KCS6, KCS11, KCS12, and KCS20) formed a single subclade, respectively (Fig. 5). Thus, we speculated that the *evm.TU.CTG175.54* might be a new *Kcs* gene during fatty acid elongation under drought stress in *Pse. libanotica* and named it as *PlKCS5/6.*

**Fig. 6:**
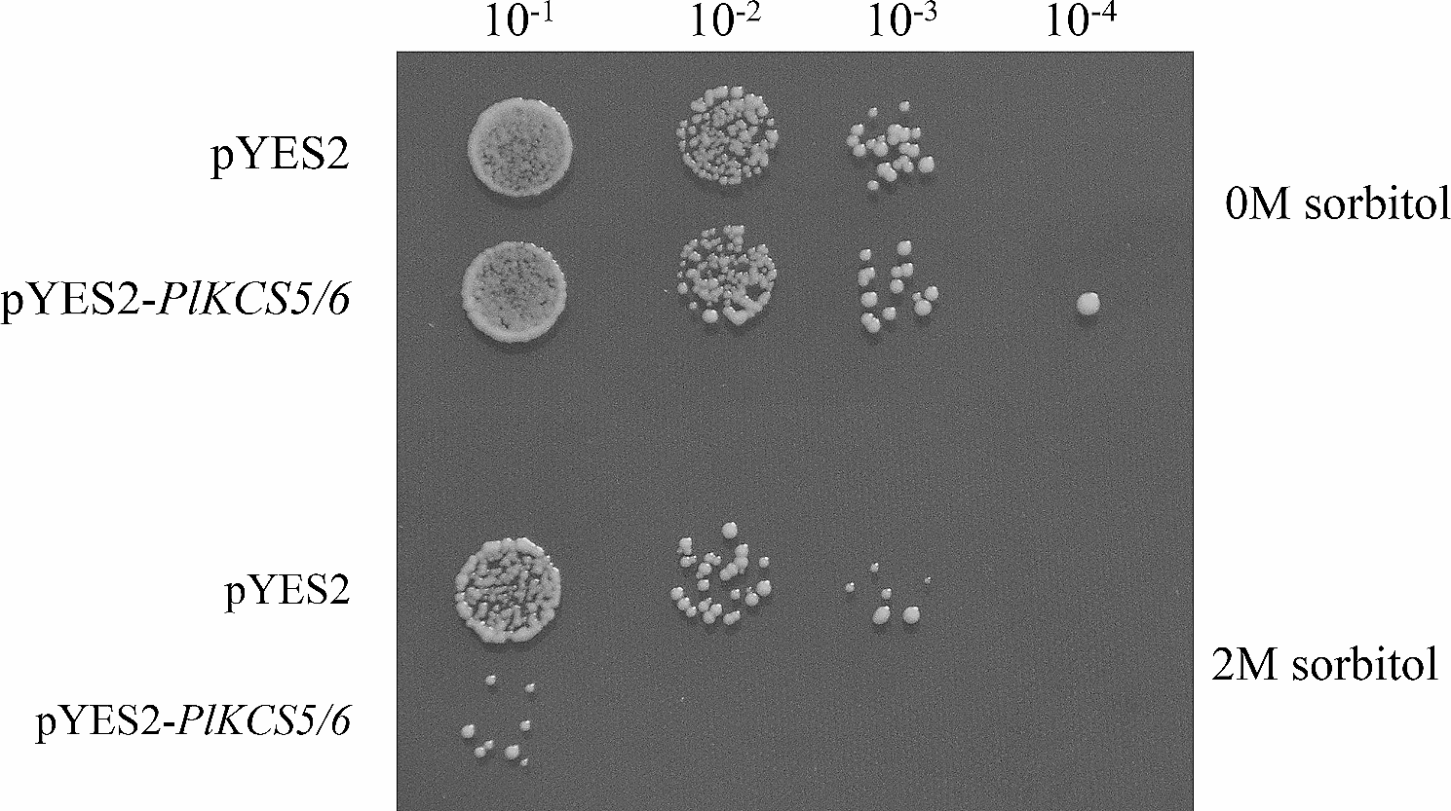
The growth activity of pYES2 and pYES2-*PlKCS5/6* under different treatments. 5 µL of serial dilutions of pYES2 and pYES2-*PlKCS5/6* stressed with 0 M sorbitol and 2 M sorbitol were cultivated on SC-Ura medium for 2 days

To investigate the growth of yeast cells exposed to stresses, the yeast cells harboring empty pYES2 (as a control) or pYES2-*PlKCS5/6* plasmid was evaluated after drought treated with 2 M sorbitol (Fig. 6). The results showed that the growth rate of yeast contained empty pYES2 and pYES2-*PlKCS5/6* had no difference under the treatment of 0 M sorbitol. However, the growth of the PYES2-*PlKCS5/6* plasmid was worse than empty pYES2 under 2 M sorbitol treatment (Fig. 6). These results demonstrated that expression of the *PlKCS5/6* gene in yeast related to the abiotic stress treatments. The function of *PlKCS5*/*6* needs further in-depth research.

## Discussion

### Characterization of the reference genome of *pse. Libanotica*

Karyotype comparative analysis displayed that the chromosome length of the St genome is shorter than other genomes in Triticeae, such as R genome (*Secale*), H genome (*Hordeum*), P genome (*Agropyron*), Ns genome (*Psathyrostachys*), and E genome (*Thinopyrum*) [34]. In this study, we assemble a highly contiguous reference genome sequence of *Pse. libanotica* with 2.99 Gb in size, by using Illumina sequencing, nanopore-based next-generation sequencing, and chromosome-scale scaffolding by Hi-C. The genome size of St is smaller than other diploid Triticeae species reported at present, such as the D genome (4.3 Gb) of *A. tauschii* [35], the E genome (4.78 Gb) of *Th. elongata* [36], and the R genome (7.74 Gb) of *S. cereale* [37]. Repeat sequences comprise 71.62% of the assembled *Pse. libanotica* genome which is lower than repetitive proportions in *Hordeum vulgare*, *Th. elongatum*, *Triticum aestivum*, *Aegilops speltoides* and *Secale cereal* (varies from 80.8 to 90.31%) even though they displayed no composition difference. Thus, we suppose that less repetitive sequence may responsible for smaller St genome size.

From the standpoint of germplasm innovation for breeding, clarifying the genome relationships in Triticeae can provide useful strategies for obtaining germplasm with high genetic diversity and fitness by artificial hybridization [3]. In this study, we found that the St genome is closely related to the A, B, and D genomes of wheat, offering the potential to transfer gene(s) from the St genome to wheat. Furthermore, we observed that *T. aestivum* 4 A was syntenic to *Pse. libanotica* chromosome 4St, 5St, and 7St, and *T. urartu* 4 A was syntenic to *Pse. libanotica* chromosome 4St and 5St, which were related to the most significant overall re-arrangement of chromosome 4 A in wheat [38, 39].

The St genome combines with other basic genomes formed more than 60% perennial Triticeae species [2, 3]. These St-containing species are excellent forages for feeding livestock and contain abundant resistant genes for crop breeding and improvement [6–9]. *Pse. libanotica* is an often-crosspollination plant, with an estimated genome size of 1.34% heterozygous. Cytogenetic research revealed that 5St and 7St displayed high-level genetic heterogeneity among seven pairs of chromosomes of *Pse. libanotica* [15]. The high genomic heterozygosity could frequently occur from open pollination plants, which not only spurs the establishment of fitness and abundant genetic diversity but also contributes to the genetic advantage in terms of the richness of their polyploid offspring species in Triticeae. Many natural hybrids between various St-containing polyploid species have been reported [40–43], further enlarging the richness of St-containing descendants.

The hierarchy of phylogenetic tree based on all single-copy gene families in this research is similar to that from few nuclear single-copy genes [10], whole chloroplast genome [3] and nuclear transcriptome sequencing [11]. However, the estimate of diverge time is slightly different. We evaluated the *Pseudorogneria* diverged 10.7 Mya (10.0 ~ 11.4 Mya), which is earlier than 8.0 Mya (5.6 ~ 10.3 Mya) based on nuclear single-copy genes, but later than 14.5 Mya (14.4 ~ 14.7 Mya) inferred from nuclear transcriptome. The difference might be caused by different genetic information used and different species used for constructing the chronogram. Yet despite all that, the speculated diverge time belongs to the middle and late Miocene (5.3 ~ 15 Mya). During Miocene stage, the ice sheet grew, and the Tethyan Seaway went through a gradually complete closure leading to the low temperature and water deficit in the Iranian Plateau [44]. The St genomic analysis revealed that unique gene families and expanded families displayed enrichment in cutin, suberine, and wax biosynthesis. Morphologically, the aerial parts of *Pse. libanotica* is covered by cuticular wax [2]. It has been reported that plant cuticular wax, which acts as the first barrier against environmental threats, consistently serves a critical role in restricting nonstomatal water loss which was often related to drought resistance [19–21, 45, 46]. Thus, it is reasonable to speculate that *Pse. libanotica* diverged during the middle and late Miocene in Tran and might develop cuticular wax resistance to cold and drought environments.

In this study, Ks was calculated in *Pse. libanotica, D. glomerat*, and *B. distachyon* to identify putative whole-genome duplication events by using each gene pair in syntenic blocks. The results indicated that a whole-genome duplication event occurred in the common ancestor of Poaceae, which is consistent with previous study [47]. However, *Pse. libanotica* and other species used for calculated Ks are heterozygous, and polymorphic positions are represented by only one of the possible nucleotides in assembled sequences. Carina et al. [48] think that traditionally methods (using a single representative DNA sequence per species) ignored the presence of polymorphisms, thus leading to bias. To address the problem, they apply a time-dependent Poisson random field model of sequence divergence to study codon evolution in protein coding genes. They suggested a best-practice protocol for the estimation of Ka/Ks and illustrate the performance of this protocol by studying genome data set of four crow species [48]. Therefore, the effect of heterozygosity or polymorphism on Ks should be fully considered in subsequent studies.

### A combination of cuticular wax and related genes might promote the excellent drought resistance of *pse. Libanotica*

Cuticular waxes mostly comprise VLCFAs and their derivatives. In this study, 14 genes involved in cuticular wax biosynthesis were identified in leaves of *Pse. libanotica* during 28 days of drought treatment. The *Acc* was reported to catalyze the first step and increased significantly the overall rate of fatty acid biosynthesis up to C16:0, C16:1, and C18:1 [49]. The long-chain fatty acids are further hydrolyzed by *Fatb*, and the FATB thioesterase gene encodes a group of enzymes with more heterogeneous substrate specificity. However, generally shows high activities towards saturated acyl-ACPs which is the major determinant of the chain length and level of saturated fatty acid found on most plant tissues [24]. In *Pse. libanotica*, *Acc* and *Fatb*, determined the *de novo* fatty acid biosynthesis, are significantly upregulated during 21 days of water deficit which might indicate its excellent drought resistance.

Based on differences in VLCFA length and degree of unsaturation, 21 KCS genes in *Arabidopsis* showed substrate specificity and organ- or tissue-specific expression patterns during fatty acid elongation [25, 50]. It is proved that *Kcs1* is an important gene and has broad substrate specificity for saturated and mono-unsaturated C16–C24 acyl CoAs formation [51]. The *Kcs20* and *Kcs2*/*DAISY* are involved in the two-carbon elongation to C22 VLCFA, and are required for cuticular wax and root suberin biosynthesis and expressed in stem epidermal peels [52]. The *Kcs6* is essential and crucial for the production of epicuticular and pollen coat lipids that are longer than C28 [53, 54]. In this study, two *Kcs5* transcripts and *evm.TU.CTG175.54* were upregulated during VLCFA elongation. It has been reported that the *Kcs5* is responsible for C26–C34 formation in *Arabidopsis* [55], whereas gene responsible for the C18 elongated to C26 is missing in *Pse. libanotica*. The *evm.TU.CTG175.54* displayed partly DNA and protein sequence similarity with published KCS5 and KCS6, and differed from other reported KCS gene families. Thus, we speculate that *evm.TU.CTG175.54* might be a new gene responsible for C18 to C26 biosynthesis during VLCFA elongation in *Pse. libanotica*.

The fatty acyl-CoA reductase (FAR) gene family is expressed in the epidermis of aerial tissues and in roots, which is responsible for the primary alcohol formatting pathway. In *Pse. libanotica*, the *Far1* was downregulated, while the *Far4* was upregulated. It has been reported that *Far4* catalyzes the reduction of VLC-acyl-CoAs to primary alcohols, presumably via an unreleased aldehyde intermediate which was produced through alkane forming pathway [56].

The *Cer1*, *Cer3*, *Cyp96a15*/*Mah1* related to alkane forming pathway were significantly upregulated in *Pse. libanotica*. *Cer1* interacts with the wax-associated protein *Cer3* and endoplasmic reticulum–localized *Cytb5s* resulting in VLC alkane synthesis [28]. The alkane was further hydrolyzed by *Cyp96a15*/*Mah1*, which led alkanes producing secondary alcohols and corresponding ketones [26].

To sum up, we assume that the aldehydes, alkanes, secondary alcohol, and ketones accumulated in the alkane forming pathway are the main cuticular wax component in *Pse. libanotica* leaves. These might increase wax deposition and restricting water loss.

## Conclusions

In this study, we reported the chromosome-level genome assembly for the St genome of *Pse. libanotica*. The total genome size is 2.99 Gb and 46,369 protein-coding genes were annotated with 71.62% repeat sequences. During the middle and late Miocene, unique genes, gene family expansion, and contraction in *Pse. libanotica* were enriched in biotic and abiotic stresses, such as fatty acid biosynthesis which may greatly contribute to its drought adaption. Furthermore, we investigated genes associated with the cuticular wax formation and water deficit and found a new *Kcs* gene *evm.TU.CTG175.54* plays a critical role in the VLCFA elongation from C18 to C26 in *Pse. libanotica*. Our study lays a foundation for the genome diversification of Triticeae species and deciphers cuticular wax formation genes involved in drought resistance.

## Materials and methods

### Plant materials

The diploid *Pse. libanotica* accession PI 228,392 (2n = 14) was used for genome sequencing in this study. The original seeds were collected from the northeast side of Kuhe Savalan, Azerbaijan, Iran (47.85 N, 38.30E). It was kindly provided by the National Plant Germplasm System (NPGS, United States). The voucher specimens were kept in the herbarium of Triticeae Research Institute, Sichuan Agricultural University, China.

## DNA extraction and library preparation

High-molecular-weight genomic DNA was extracted from fresh leaves of a single plant using a DNAsecure Plant Kit (TIANGEN, Beijing, China). For Nanopore sequencing, a ligation of the sequencing adapters library was prepared following the manufacturer’s protocol (PromethION, CA). For Illumina (San Diego, CA) short-read sequencing, libraries were size-selected for PE150 sequencing. Sequencing libraries with insert sizes of 350 bp were constructed and sequenced using an Illumina HiSeq X Ten platform at the Novogene Bioinformatics Institute, Beijing.

## Genome assembly

We constructed a *de novo* assembly of the St genome of *Pse. libanotica* PI 228,392 by combining sequences from three different technologies: Illumina PE150 short-read sequencing, Nanopore long-read sequencing, and Hi-C conformational alignment. The clean Nanopore reads after filtering and decontamination were assembled with wtdbg2 (v2.5). The error-corrected reads were aligned to each other and assembled into genomic contigs. The iterative polishing was conducted using Pilon (v1.22) in which clean Illumina reads were aligned with the pre-assembled contigs and BWA-MEM with the default parameters [57, 58]. Further, we combined the final pre-assembled contig sequences from Nanopore sequencing and clean paired-read data from Illumina sequencing into scaffolds using SSPACE (v3.0) tool [59]. Genome assembly completeness was assessed using the plantae database of 1440 single-copy orthologues using BUSCO (v3) with a BLAST threshold E-value of 1 × 10^− 5^. The LAI was used to evaluate the assembly quality [31]. GenomeTools v1.6.2 (gt ltrharvest) and LTR_Finder v1.07 was used to accurately identify LTR-RTs. LAI was calculated with parameters: -t 24 -window 3,000,000 -step 300,000 -1. Higher LAI scores correspond to more complete genome assemblies because a greater number of intact LTR retrotransposons are identified in these cases.

The Hi-C libraries were prepared as described previously [60]. Hi-C library sequence used a modified SNAP read mapper to align the draft input assembly (http://snap.cs.berkeley.edu) [61]. HiRise was used to analyze the segregation of Hi-C read pairs mapped within draft scaffolds, and a likelihood model of the genomic distance between the read pairs was generated. The model was used to identify and break putative mis-joins, score prospective joins, and select joins above a threshold.

### Annotation of repetitive sequences

Both homology-based and *de novo-*based approaches were used to search for TEs. Tandem Repeat was extracted using TRF (http://tandem.bu.edu/trf/trf.html) by ab initio prediction. The homolog prediction used Repbase (http://www.girinst.org/repbase) database employing RepeatMasker (http://www.repeatmasker.org/) software and its in-house scripts (RepeatProteinMask) with default parameters to extract repeat regions. For the *de novo*-based approach, we used LTR_FINDER (http://tlife.fudan.edu.cn/ltr_finder/), RepeatScout (http://www.repeatmasker.org/), and RepeatModeler (http://www.repeatmasker.org/RepeatModeler.html) to build the *de novo* repeat library. All the repeats identified by different methods were combined into the final repeat annotation after removing the redundant repeats.

### Genome annotation

To predict protein-coding genes, three approaches were used: *de novo* gene prediction, homolog prediction, and RNA-sequencing annotation. For *de novo* prediction, Augustus (v3.2.3), Geneid (v1.4), Genescan (v1.0), GlimmerHMM (v3.04), and SNAP (http://homepage.mac.com/iankorf/) were applied to predict genes. For homolog prediction, the protein sequences of twelve published plant genomes (*A. tauschii*, *B. distachyon*, *T. aestivum*, *T. durum*, *T. dicoccoides*, *T. urartu*, *H. vulgare*, *O. sativa*, *S. cereale*, *Sorghum bicolor*, *Z. mays*, and *Arabidopsis thaliana*) were aligned to the genome using TblastN (v2.2.26; E-value ≤ 1e-5), and then used Gene-Wise (v2.4.1) [62] to predict gene structures. To optimize the genome annotation, the RNA-seq reads were aligned to the genome using TopHat (v2.0.11), and the alignments were used as input for Cufflinks (v.2.2.1) [63, 64]. The non-redundant reference gene set was generated by merging genes predicted by three methods with EvidenceModeler (v1.1.1) using PASA (Program to Assemble Spliced Alignment) terminal exon support and including masked transposable elements as input into gene prediction [65].

Genes functions were assigned according to the best match by aligning the protein sequences to the Swiss-Prot (with a threshold of E-value ≤ 1e^-5^) [66]. The motifs and domains were annotated using InterProScan70 (v5.31) by searching against publicly available databases, including ProDom, PRINTS, Pfam, SMRT, PANTHER, PROSITE, and eggNOG (v5.0) [67–71]. The Gene Ontology (GO) IDs for each gene were assigned according to the corresponding InterPro entry.

### Constructing gene families

To construct the dataset for gene-family clustering, the protein sequences from the genomes of *Pse. libanotica* and 13 other plants (*A. tauschii* [35], *B. distachyon* [72], *T. aestivum* [73], *T. durum* [74], *T. dicoccoides* [75], *T. urartu* [76], *H. vulgare* [77], *O. sativa* [78], *S. cereale* [37], *Sorghum bicolor* [79], *Z. mays* [80], *Dactylis glomerata* [81] and *Arabidopsis thaliana* [82] were used. In the included species, only the longest transcript in the coding region was retained for further analysis when multiple transcripts were present in a gene. Additionally, genes encoding proteins with fewer than 50 amino acids were filtered. The protein sequences of all species were filtered by BLASTP with an E-value of 1e^-5^ Protein sequences from all 14 species were clustered into paralogous and orthologous groups using OrthoMCL (http://orthomcl.org/orthomcl/) with an inflation parameter equal to 1.5.

### Phylogenetic tree reconstruction

Protein sequences of all single-copy gene families were aligned using MUSCLE [83], and the alignments of each gene family were concatenated into a super-alignment matrix. These data matrices were used for maximum likelihood phylogenetic analyses by RAxML (http://sco.h-its.org/exelixis/web/software/raxml/index.html) with a bootstrap value of 100, where *A. thaliana* was designated as the outgroup. The Venn diagram was constructed to display the number of gene families that were shared among four Poaceae species (*A. tauschii*, *T. aestivum*, *H. vulgare*, and *S. cereale*) clustered into one group of the phylogenetic tree.

### Species divergence time estimation

Single-copy gene families among *Pse. libanotica*, *(A) tauschii*, *(B) distachyon*, *T. aestivum*, *T. durum*, *T. dicoccoides, T. urartu*, *H. vulgare*, *O. sativa*, *S. cereale*, *Sorghum bicolor*, *Z. mays*, *Dactylis glomerata* and *Arabidopsis thaliana* were selected using the MCMCTree program (http://abacus.gene.ucl.ac.uk/software/paml.html) in phylogenetic analysis with Maximum Likelihood (PAML) for an estimate the divergence time of the nodes on the phylogenetic tree. The MCMCTree parameters were as follows: a burn-in of 10,000 steps, a sample number of 100,000, and a sample frequency of 2. The calibration times of divergence were obtained from the TimeTree database (http://www.timetree.org/): 2.20–3.80 Mya for *T. durum* and *T. aestivum*, 3.60–4.40 Mya for *T. dicoccoides* and *T. urartu*, 10.0–11.40 Mya for *Pse. libanotica* and *(A) tauschii*, 9.60–11.90 Mya for *S*. c*ereale* and *H. vulgare*, 31.4–47.2 Mya for *Dactylis glomerata* and *(B) distachyon*, 9. 0–12.0 Mya for *S. bicolor* and *Z. mays*, 47.2–189 Mya for *O. sativa* and *A. thaliana*.

### Gene family expansion and contraction

The expansion and contraction of gene families were determined by comparing the cluster size differences between the ancestor and each species using the CAFE program (http://sourceforge.net/projects/cafehahnlab/). A random birth-and-death model was used to evaluate changes in gene families of the phylogenetic tree. A probabilistic graphical model (PGM) was used to calculate the transfer probability of each gene family from parent to child nodes in the phylogeny. The conditional likelihood was used as the test statistics to calculate the corresponding *P*-value of each lineage, and a *P*-value of or below 0.05 was considered significant.

### Whole-genome duplication

The homologous search in the *Pse. libanotica* genome was performed using BLASTP (E-value < 1e^-5^), and then MCScanX (http://chibba.pgml.uga.edu/mcscan2/) was used to identify syntenic blocks in the genome according to the gene location and blast results. For each gene pair in syntenic blocks, Ks values were calculated, and values of all gene pairs were plotted to identify putative whole-genome duplication events in *Pse. libanotica*.

### Drought experiments

The seeds of *Pse. libanotica* PI 228,392 were germinated in a petri dish lined with a double layer of filter paper. Afterward, young seedlings were transplanted to potted monocultures and cultivated in a greenhouse at a temperature of 20℃ with a light cycle of 16 h/8 h until the sturdy stage and had a certain number of tillers.

For drought treatments, adult stage (five-month-old) potted *Pse. libanotica* plants of uniform growth were selected for water deficit treatment. The experimental groups were not watered for 28 days (every seven days were taken as drought stress 7d, 14d, 21d, 28d, and each treatment has three biology repeats). The control group was watered every seven days during 28 days experiments. Fresh leaves of each treatment were taken at 9–10 am and stored at -80℃.

### Library preparation, transcriptome sequencing and analysis

Accurate detection of RNA integrity and total volume with Agilent 2100 bioanalyzer. NEB general library building using NEBNext® Ultra™ RNA Library Prep Kit for Illumina® kit and strand-specific library building using NEBNext® Ultra™ Directional RNA Library Prep Kit for Illumina® kit. After passing the library test, the different libraries are pooled according to the effective concentration and the target downstream data volume required for Illumina sequencing. The basic principle of sequencing is sequencing while synthesizing. The library construction and Illumina sequencing were conducted at Novogene limited liability company (Beijing, China).

Clean data were obtained after quality clipping of the raw data and Q20, Q30, and GC content. All the downstream analyses were based on clean data with high quality. We used the assembly *Pse. libanotica* as the reference genome. Reads were aligned to the reference genome using Hisat2 (v2.0.5) which can generate a database of splice junctions based on the gene model annotation file and thus produce a better mapping result than other non-splice mapping tools. The mapped reads of each sample were assembled by StringTie (v1.3.3b) in a reference-based approach [84]. FeatureCounts v1.5.0 was used to count the reads numbers mapped to each gene. And the FPKM values were then mapped back to read counts according to known gene lengths. Differential expression analysis of two conditions/groups was performed using the DESeq2 R package (1.20.0). The resulting P-values were adjusted using the Benjamini and Hochberg’s approach for controlling the false discovery rate.

Differentially expressed genes (DEGs) were selected using criteria| log2FC| ≥ 1 and adjusted q-value < 0.05. We plot the venn diagram by the number of differential expressed genes in each experiment group. All numbers in the circle represent the total different genes in the comparison groups, the overlapping regions indicate different genes between groups. All DEGs were mapped to individual terms in the gene ontology (GO) database and the number of genes per term was calculated by the clusterProfiler R package. Analysis of gene regulatory pathways was conducted using the KEGG pathway database [85, 86]. Finally, based on the above analysis, wax biosynthesis genes and *Kcs* genes were obtained from these DEGs.

### Electronic supplementary material

Below is the link to the electronic supplementary material.

Supplementary Material 1

Supplementary Material 2

Supplementary Material 3

Supplementary Material 4

Supplementary Material 5

Supplementary Material 6

Supplementary Material 7

Supplementary Material 8

Supplementary Material 9

Supplementary Material 10

Supplementary Material 11

Supplementary Material 12

Supplementary Material 13

Supplementary Material 14

## Data Availability

All data are available in the manuscript, the supplementary materials, or at publicly accessible repositories. These data in the public repositories include all raw reads and assembled sequence data for *Pse. libanotica* in NCBI under BioProjectID PRJNA940619, Nanopore sequencing data (SAMN33575612), Illumina sequencing data (SAMN33589869), Hi-C data (SAMN34127759), and RNA-seq data (SAMN33846863, SAMN33593293). The assembly and annotation data of *Pse. libanotica* in the Genome Warehouse in BIG Data Center under accession numbers WGS038485, which are accessible at https://bigd.big.ac.cn/gwh.
