# Supplementary material for: A chromosome level genome assembly of Pseudoroegneria Libanotica reveals a key Kcs gene involves in the cuticular wax elongation for drought resistance
Source: BMC Genomics. 2024 Mar 6;25:253. doi: 10.1186/s12864-024-10140-5 (PMC10916072; doi:10.1186/s12864-024-10140-5)
Supplement: Supplementary file 1 — Supplementary Material 1 [file 12864_2024_10140_MOESM1_ESM.pdf]

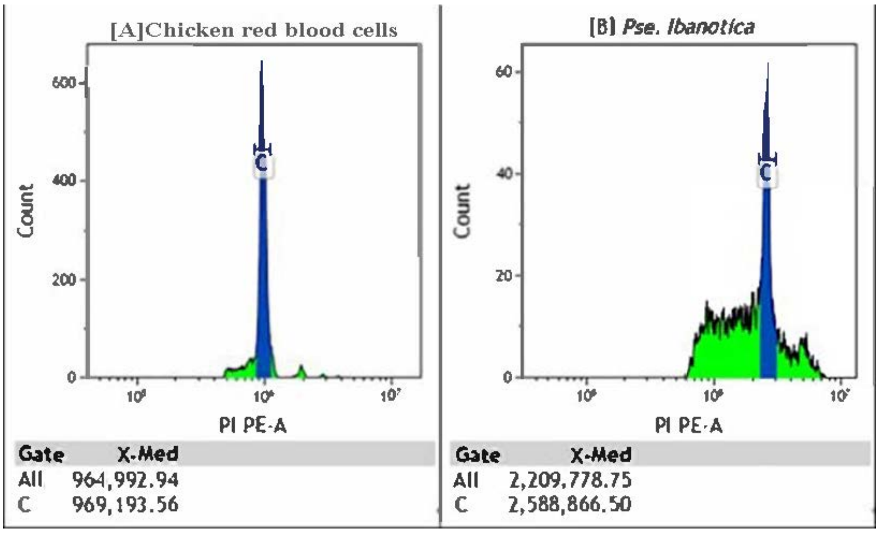

Fig. S1 Flow cytometry results of *Pse. libanotica*

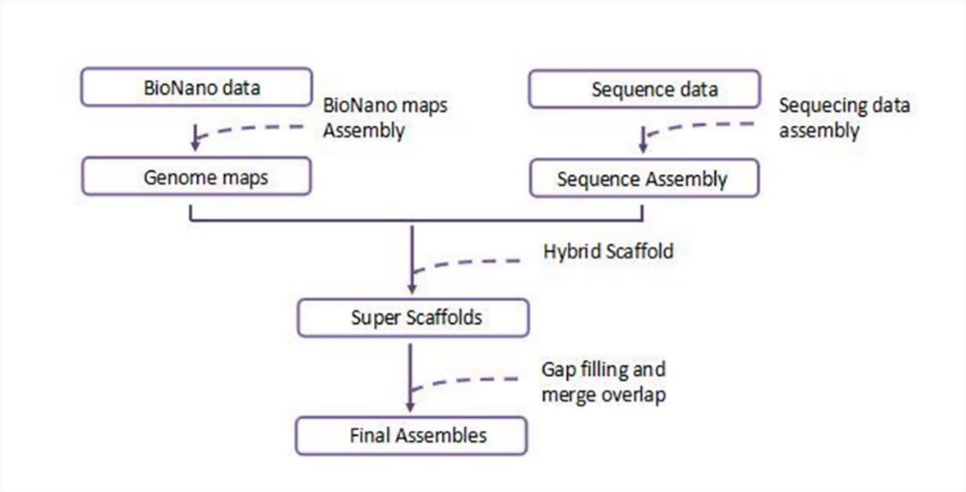

Fig. S2 The workflow of *Pse. libanotica* genome assembling

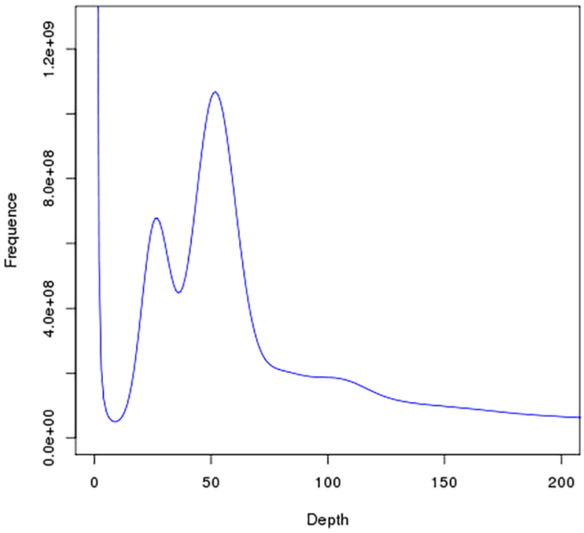

Fig. S3 K-mer frequency distributions in *Pse. libanotica.* Axis means sequence depth (X), and y axis means frequency of K-mer

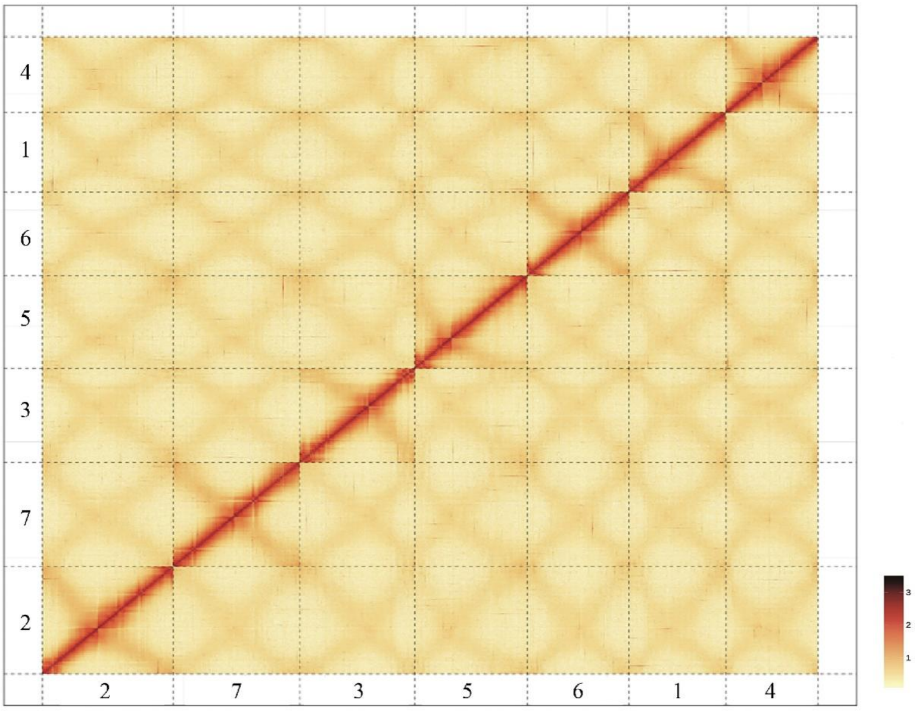

Fig. S4 Hi-C interaction matrix for genome assembly. The x and y axes indicate the mapping positions of the first and second read in the read pair respectively, grouped into bins. The color of each square gives the number of read pairs within that bin. Scaffolds less than 1 Mb are excluded.

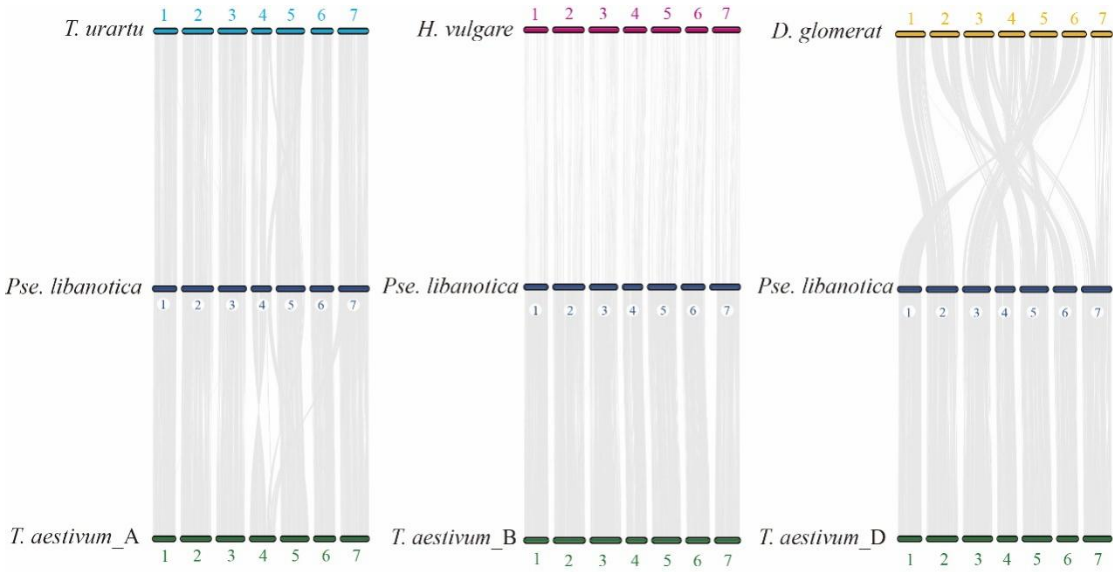

Fig. S5 Synteny analysis of seven chromosomes from *Pse. libanotica* with *T. aestivum*, *T. urartu*, *H. vulgare*, and *D. glomerat*

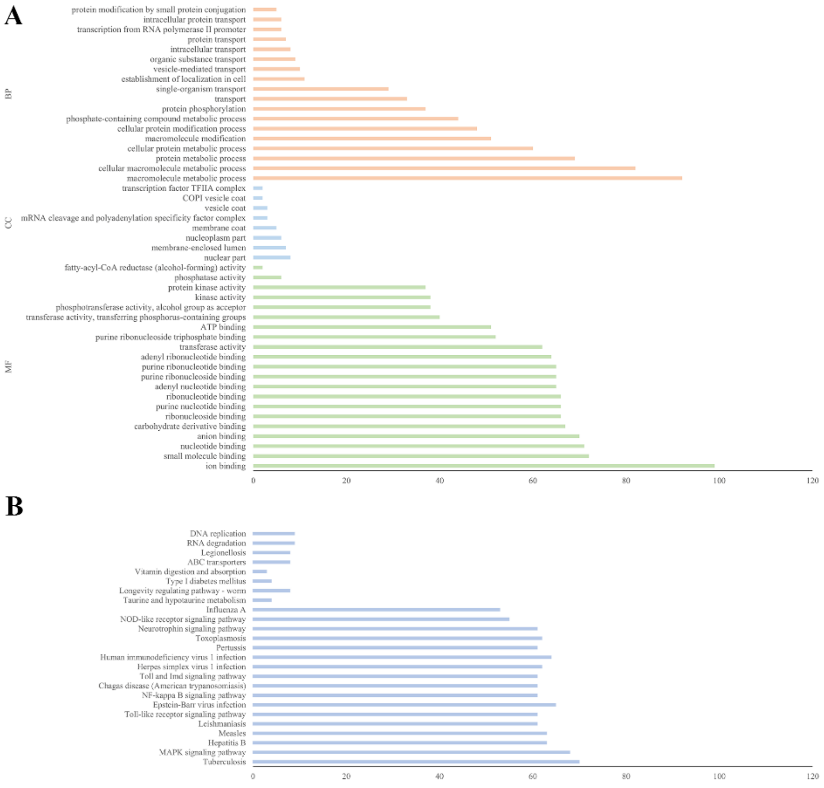

Fig. S6 Fig.S6 GO (A) and KEGG (B) pathway of unique families in *Pse. libanotica*.

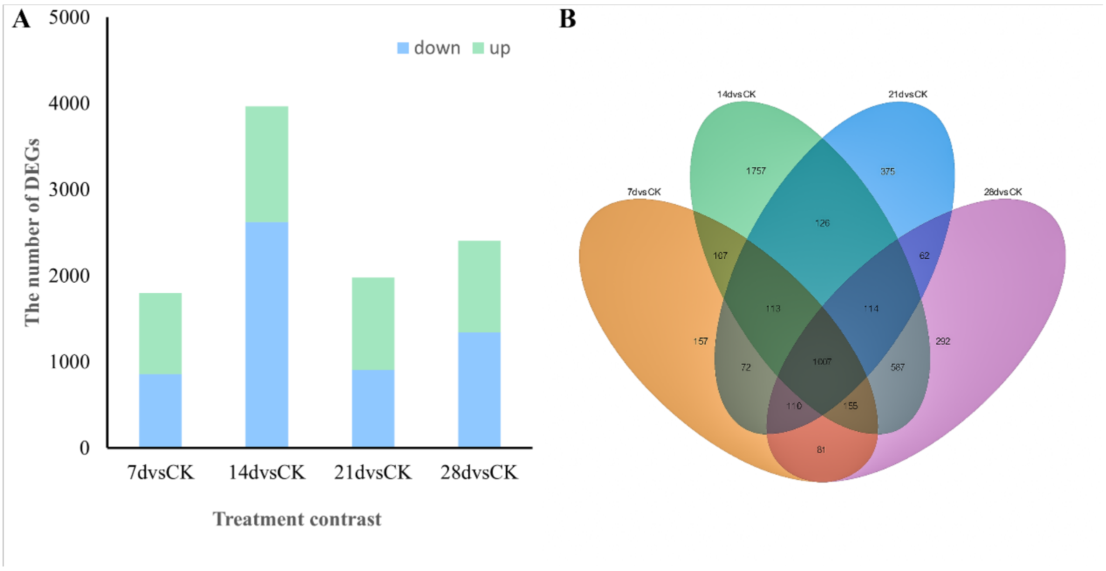

Fig. S7 Number of differentially expressed genes (DEGs) under drought conditions at 7d, 14d, 21d and 28d. Venn diagrams showing the number of co-expressed DEGs in *Pse. libanotica* under drought stress.

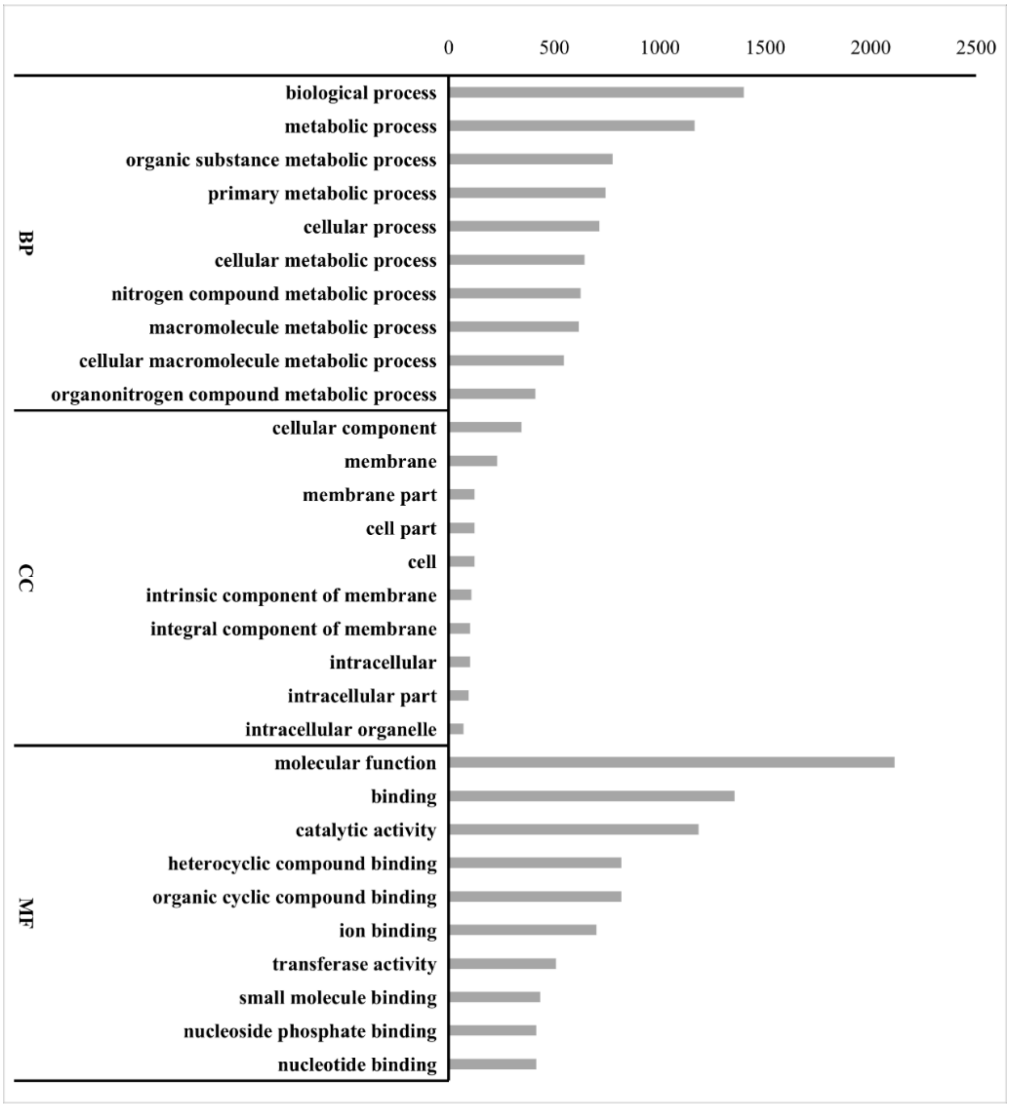

Fig. S8 GO classification of differentially expressed genes (DEGs) of *Pse. libanotica* under drought stress. The ordinate represents GO term, and the abscissa indicates the gene number enriched in GO term.

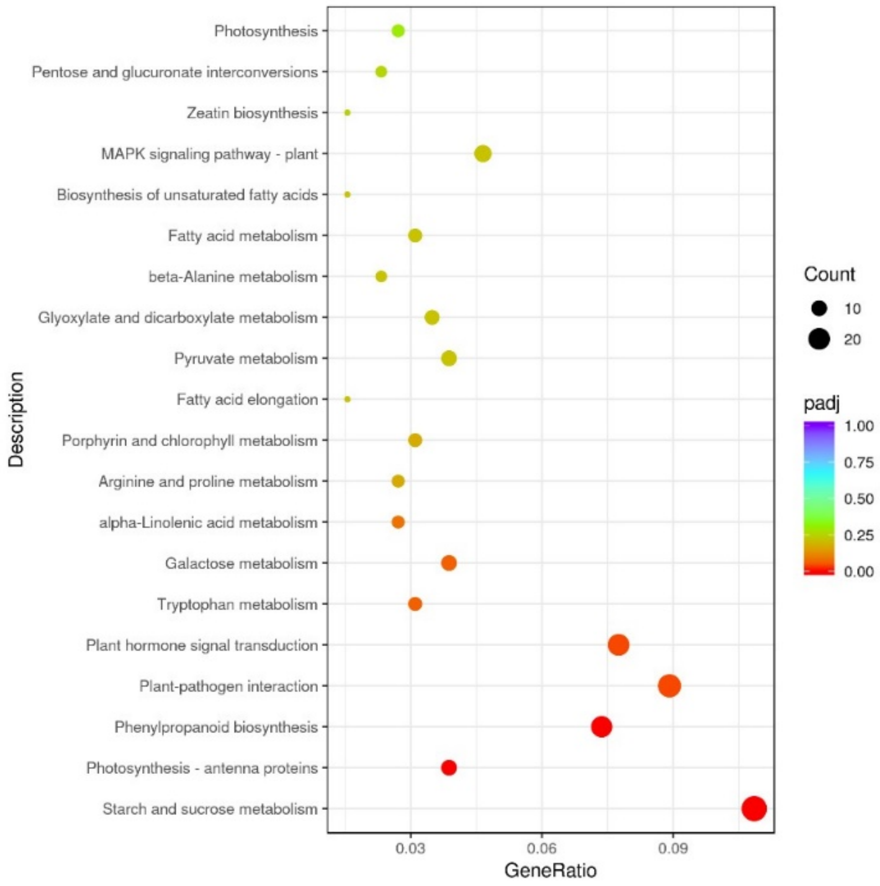

Fig. S9 KEGG pathway enrichment scatter diagram of DEGs. The19 most strongly represented pathways are displayed in the diagram. The degree of KEGG pathway enrichment is represented by the GeneRatio, the padj, and the number of genes enriched in a KEGG pathway.

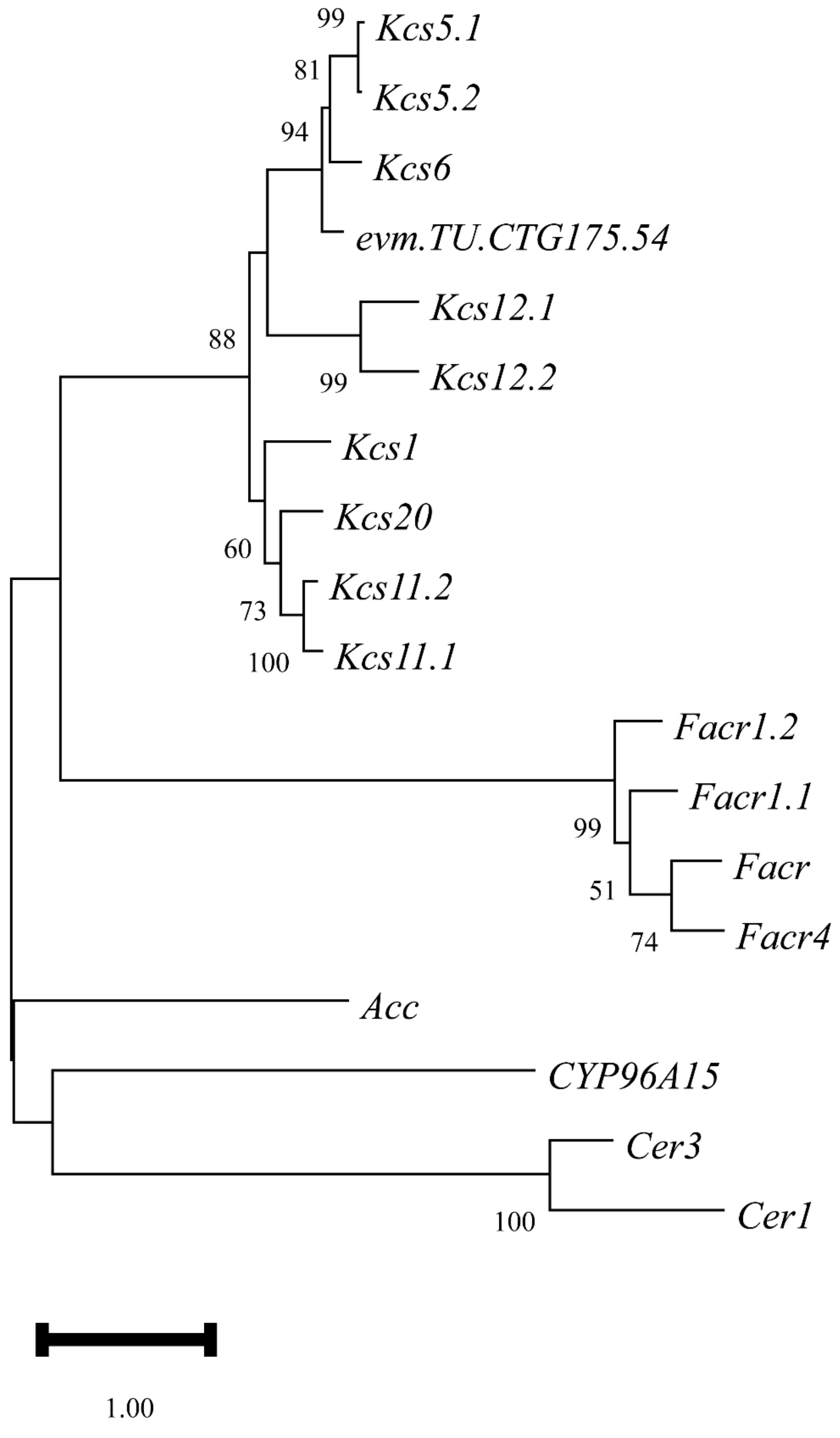

Fig. S10 Maximum likelihood tree derived from 18 candidate genes involving fatty acid biosynthesis.
