# Supplementary material for: A chromosome level genome assembly of Pseudoroegneria Libanotica reveals a key Kcs gene involves in the cuticular wax elongation for drought resistance
Source: BMC Genomics. 2024 Mar 6;25:253. doi: 10.1186/s12864-024-10140-5 (PMC10916072; doi:10.1186/s12864-024-10140-5)
Supplement: Supplementary file 2 — Supplementary Material 2 [file 12864_2024_10140_MOESM2_ESM.pdf]

Supplementary Table 1 Estimation of genome size

K-mer	Depth	n_K-mer	Genome size(Mb)	Revise Genome size(Mb)	Heteozygous rate(%)	Repeat rate(%)
19	51	157,918,766,016	3094.45	3,048	1.34	71

Supplementary Table 2 Sequencing libraries and statistics of the data used for the genome assembly

Pair-end libraries	Insert size	Total data (G)	Read length (bp)	Sequence coverage (X)
Illumina reads	350bp	191	150.00	63.67
Nanopore	--	440.60	--	146.87
Total	--	631.60	--	210.54

Supplementary Table 3 Characteristics of *Pse. libanotica* assembly containing 7 chromosome.

Chromosome name	Chromosome length (bp)	number of genes
chr1	343,212,279	5419
chr2	464,035,102	7220
chr3	408,139,130	6307
chr4	327,295,031	4534
chr5	398,549,212	6504
chr6	362,840,832	5344
chr7	449,314,708	6898
un	236,383,112	4143
total	2,989,769,406	46369

Supplementary Table 4 QV value in the *Pse. libanotica* genor

Chr	ue kmer in ge	in genome and	QV	error rate
Chr1	35510771	343145998	22.8496	0.005188
Chr2	42946485	463956002	23.3585	0.004615
Chr3	37787741	408066087	23.3567	0.004617
Chr4	33167603	327239628	22.9448	0.005076
Chr5	38520265	398484011	23.161	0.004829
Chr6	33859959	362777071	23.3208	0.004655
Chr7	42003744	449230808	23.3127	0.004664

Supplementary Table 5 Evaluation of Benchmarking Universal Single-Copy Orthologs (BUSCO) and gene space coverage using core eukaryotic gene mapping approach (CEGMA) in *Pse. libanotica* genome

Species	Size (Mb)	BUSCO	CEGMA			
			Complete		Complete + partial	
			Prots	Completeness (%)	Prots	Completeness (%)
Pse. libanotica	2.99Gb	C:95.2%[S:90.1%,D:5.1%],F:1.2%,M:3.6%,n:1440	228	91.94	239	96.37

Note:

Size: genome size.

BUSCO notation: C:Complete BUSCOs;S: Complete and single-copy BUSCOs;D: Complete and duplicated BUSCOs; F: Fragmented BUSCOs;M: Missing BUSCOs;n:Total BUSCO groups searched.

CEGMA notation: Complete: Over 70% assembled core genes.

CEGMA notation: Complete + partial: Partial assembled core gene; Prots: the number of assembled genes; %completeness: the proportion of assembled core gene to core gene from the database.

Supplementary Table 6 Statistics of short high-quality reads mapping

		Percentage
Reads	Mapping rate (%)	98.95
Genome	Average sequencing depth	58.95

Supplementary Table 7 Assessment of *Pse. libanotica* genome using full length EST sequences

	Dataset	Number	with >90% sequence in one scaffold		with >50% sequence in one scaffold	
			Number	Percent (%)	Number	Percent (%)
Version	>200bp	151,872	132,240	87.073	145,315	95.683
	>500bp	54,907	47,838	87.126	53,048	96.614
	>1000bp	25,517	22,361	87.632	24,824	97.284
	>2000bp	9,212	7,952	86.322	8,958	97.243

Supplementary Table 8 Prediction of protein-coding genes in *Pse. libanotica*

Gene set		Number	Average gene length (bp)	Average CDS length (bp)	Average exon length (bp)	Average intron length (bp)	Average exons per gene
De novo	Augustus	110,674	1809.41	819.70	304.51	584.98	2.69
	GlimmerHMM	464,723	5372.95	498.87	246.18	4748.53	2.03
	SNAP	260,231	3468.79	530.63	215.50	2009.27	2.46
	Genscan	207,833	6326.56	558.07	190.11	2980.25	2.94
	Geneid	352,062	2482.82	523.46	213.69	1351.61	2.45
**Homolog**	*Zea mays*	45,820	2739.85	1013.98	295.85	711.00	3.43
	Secale cereale	83,117	1580.19	713.71	294.24	607.80	2.43
	Triticum aestivum	83,198	2234.77	847.11	284.41	701.37	2.98
	Aegilops tauschii	82,752	1922.42	799.69	286.07	625.34	2.80
	Sorghum bicolor	47,656	2656.60	1011.62	293.41	672.04	3.45
	Triticum dicoccoides	71,014	2101.95	815.51	273.96	650.79	2.98
	Brachypodium distachyon	58,702	2428.98	950.70	296.40	669.67	3.21
	Triticum urartu	71,014	2101.95	815.51	273.96	650.79	2.98
	Triticum durum	74,755	2308.66	851.23	3.04	279.58	712.79
	Oryza sativa	56,132	2256.06	906.08	305.03	685.11	2.97
	Hordeum vulgare	87,149	1627.20	718.52	279.53	578.60	2.57
**RNA-seq**	Transcripts	57,435	6023.16	1828.92	346.18	979.24	5.28
	PASA	109,908	3011.48	953.43	227.31	644.28	4.19
**EVM**		163,236	2106.65	721.50	286.36	911.55	2.52
**PASA-update**		162,902	2133.02	722.61	287.29	930.80	2.52
**Final set**		102,333	2676.51	845.90	284.13	925.87	2.98
