# Supplementary material for: A chromosome level genome assembly of Pseudoroegneria Libanotica reveals a key Kcs gene involves in the cuticular wax elongation for drought resistance
Source: BMC Genomics. 2024 Mar 6;25:253. doi: 10.1186/s12864-024-10140-5 (PMC10916072; doi:10.1186/s12864-024-10140-5)
Supplement: Supplementary file 3 — Supplementary Material 3 [file 12864_2024_10140_MOESM3_ESM.pdf]

Supplementary Information

Fig. S1 Flow cytometry results of *Pse. libanotica*

Fig. S2 The workflow of *Pse. libanotica* genome assembling

Fig. S3 K-mer frequency distributions in *Pse. libanotica.* Axis means sequence depth (X), and y axis means frequency of K-mer

Fig. S4 Hi-C interaction matrix for genome assembly. The x and y axes indicate the mapping positions of the first and second read in the read pair respectively, grouped into bins. The color of each square gives the number of read pairs within that bin. Scaffolds less than 1 Mb are excluded.

Fig. S5 Synteny analysis of seven chromosomes from *Pse. libanotica* with *T. aestivum*, *T. urartu*, *H. vulgare*, and *D. glomerat*

Fig. S6 GO (A) and KEGG (B) pathway of unique families in *Pse. libanotica*

Fig. S7 Number of differentially expressed genes (DEGs) under drought conditions at 7d, 14d, 21d and 28d. Venn diagrams showing the number of co-expressed DEGs in *Pse. libanotica* under drought stress.

Fig. S8 GO classification of differentially expressed genes (DEGs) of *Pse. libanotica* under drought stress. The ordinate represents GO term, and the abscissa indicates the gene number enriched in GO term.

Fig. S9 KEGG pathway enrichment scatter diagram of DEGs. The19 most strongly represented pathways are displayed in the diagram. The degree of KEGG pathway enrichment is represented by the GeneRatio, the padj, and the number of genes enriched in a KEGG pathway.

Fig. S10 Maximum likelihood tree derived from 18 candidate genes involving fatty acid biosynthesis

Table S1 Estimation of genome size

Table S2 Sequencing libraries and statistics of the data used for the genome assembly

Table S3 Characteristics of *Pse. libanotica* assembly containing 7 chromosome

Table S4 QV value in the *Pse. libanotica* genome assembly

Table S5 Evaluation of benchmarking universal single-copy orthologs (BUSCO) and gene space coverage using core eukaryotic gene mapping approach (CEGMA) in *Pse. libanotica* genome

Table S6 Statistics of short high-quality reads mapping

Table S7 Assessment of *Pse. libanotica* genome using full length EST sequences

Table S8 Prediction of protein-coding genes in *Pse. libanotica*

Table S9 RNA annotation information

Table S10 Non-coding RNAs in the assembly of *Pse. libanotica*

Table S11 Statistics of repeat sequence in *Pse. libanotica* genome via different methods

Table S12 GO analysis for the unique gene families in *Pse. libanotica*

Table S13 KEGG pathway of unique families in *Pse. libanotica*

Table S14 Expanded and contracted families in *Pse. libanotica*

Table S15 GO analysis for the expanded gene families in *Pse. libanotica*

Table S16 KEGG pathway of expanded families in *Pse. libanotica*

Table S17 Reads and mapping ratio of RNA sequencing data under 28 days of drought stress in *Pse. libanotica*

Table S18 Co-expressed DEGs at four treatments

Table S19 Fatty acid biosynthesis candidate genes under 28 days of drought stress in *Pse. libanotica*
